# When obesity treatment goes too far: nutritional and endoscopic management of bariatric surgery complications

**DOI:** 10.1055/a-2061-7173

**Published:** 2023-04-21

**Authors:** Enad Dawod, Malorie Simons, Sanad Dawod, Neela Easwar, Nicole Cornet, Reem Z. Sharaiha, Kartik Sampath

**Affiliations:** Division of Gastroenterology and Hepatology, Weill Cornell Medicine, New York, New York, United States

Bariatric surgery is associated with complications that can be refractory to intervention in malnourished patients. We present a patient with a gastrocutaneous fistula, which occurred as a complication of Roux-en-Y gastric bypass and was initially refractory to endoscopic closure but resolved after nutritional optimization.

A 55-year-old woman who previously underwent Roux-en-Y gastric bypass was admitted owing to the inability to tolerate oral nutrition. She had a body mass index (BMI) of 13 kg/m ^2^ . She was started on total parenteral nutrition and underwent percutaneous endoscopic gastrostomy (PEG) placement into the remnant stomach, which was complicated by high-volume leak. Esophagogastroduodenoscopy (EGD) revealed a gastrogastric fistula between the remnant and excluded stomachs, a large gastrocutaneous fistula, and a narrowed pylorus ( Fig. 1 ). A fully covered metal stent was placed through the pylorus, the gastrocutaneous fistula was sutured externally with PEG removal, and an AXIOS stent (Boston Scientific, Marlborough, Massachusetts, USA) was placed across the gastrogastric fistula ( Fig. 2 ). However, the high-volume gastrocutaneous leak recurred. Repeat EGD showed that the pyloric stent had migrated distally ( Fig. 3 ). The gastrocutaneous fistula was closed luminally with X-tack sutures, and an AXIOS stent was placed across the nearly obstructed gastrojejunal anastomosis to promote gastrocutaneous fistula healing. Large-volume gastrocutaneous leakage recurred again. A multidisciplinary team recommended nutritional optimization.

**Fig. 1:**
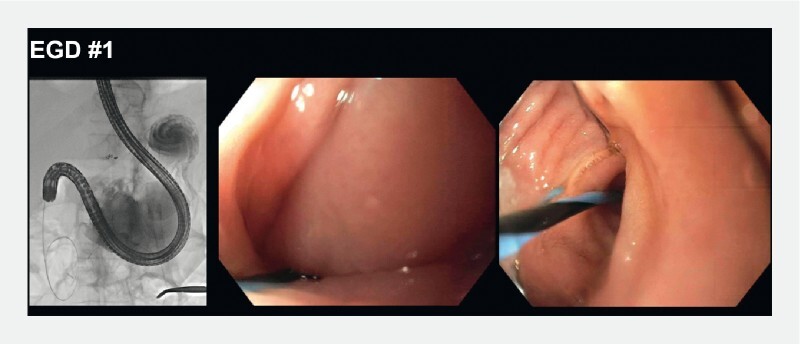
The first esophagogastroduodenoscopy (EGD) was performed to define the anatomy more clearly. It showed severe pyloric stenosis and angulation.

**Fig. 2:**
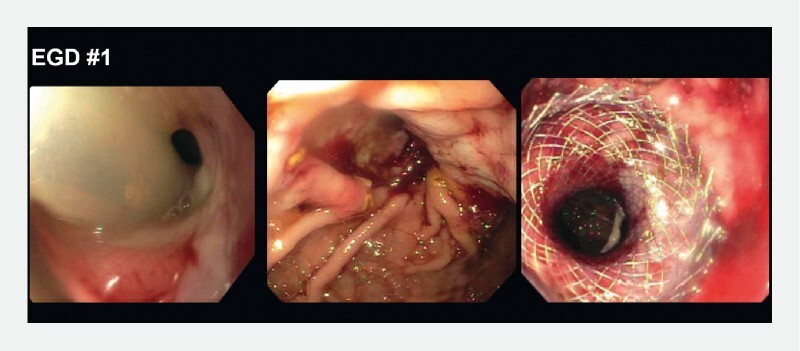
A 15 mm AXIOS stent (Boston Scientific, Marlborough, Massachusetts, USA) was placed through the angulated pylorus to maintain patency. EGD, esophagogastroduodenoscopy.

**Fig. 3:**
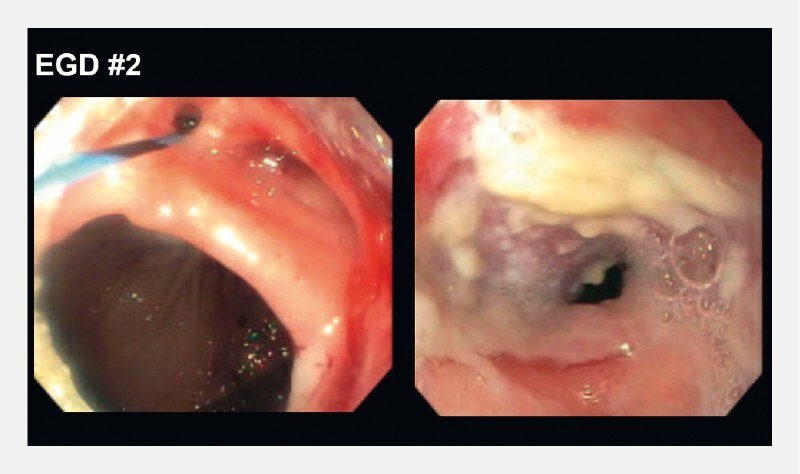
The second esophagogastroduodenoscopy (EGD) showed a migrated transpyloric stent. A large gastrocutaneous fistula was also identified.

Double-channel suturing with the OverStitch device (Apollo Endosurgery, Inc., Austin, Texas, USA) was utilized to achieve closure of the fistula, and a nasojejunal tube was placed through the gastrojejunal stent ( Fig. 4 ). The patient’s BMI ultimately increased to 20.6 kg/m ^2^ and the gastrocutaneous fistula resolved.

**Fig. 4:**
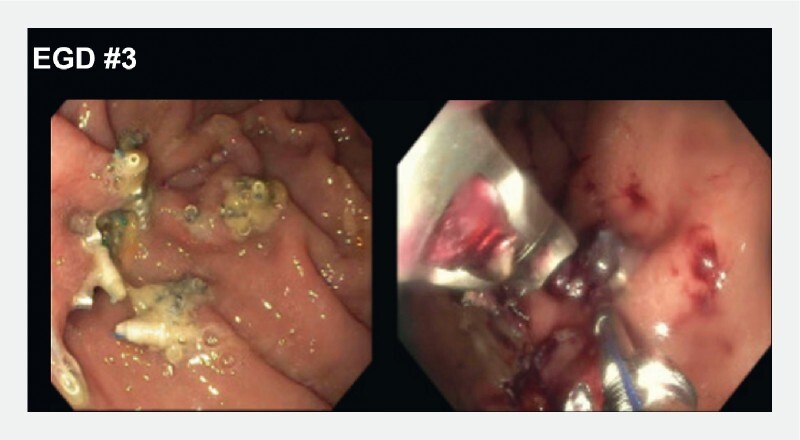
A third esophagogastroduodenoscopy (EGD) was performed following fistula recurrence. Double-channel suturing with the OverStitch device (Apollo Endosurgery, Inc., Austin, Texas, USA) was utilized to achieve closure of the fistula.

Patients with complications of bariatric surgery are often poor surgical candidates owing to malnutrition, which also hinders endoscopic repair. This case demonstrates how advanced endoscopic techniques ( Video 1 ) enabled nutritional optimization, fistula resolution, and enteral feeding via the gastrojejunal anastomosis with preservation of the Roux-en-Y anatomy. Patients with severe post-bariatric surgery complications require a multidisciplinary effort of gastroenterologists, surgeons, and nutritionists for optimal recovery.

**Video 1** Multi-step endoscopic management of a large gastrocutaneous fistula.


Endoscopy_UCTN_Code_CPL_1AH_2AG

## Notes

Endoscopy E-Videos

https://eref.thieme.de/e-videos
*E-Videos* is an open access online section of the journal *Endoscopy* , reporting on interesting cases and new techniques in gastroenterological endoscopy. All papers include a high-quality video and are published with a Creative Commons CC-BY license. Endoscopy E-Videos qualify for HINARI discounts and waivers and eligibility is automatically checked during the submission process. We grant 100% waivers to articles whose corresponding authors are based in Group A countries and 50% waivers to those who are based in Group B countries as classified by Research4Life (see: https://www.research4life.org/access/eligibility/ ). This section has its own submission website at https://mc.manuscriptcentral.com/e-videos

